# Opportunities for Outpatient Pharmacy Services for Patients with Cystic Fibrosis: Perceptions of Healthcare Team Members

**DOI:** 10.3390/pharmacy7020034

**Published:** 2019-04-03

**Authors:** Olufunmilola Abraham, Ashley Morris

**Affiliations:** Social and Administrative Sciences Division, University of Wisconsin-Madison School of Pharmacy, Madison, WI 53705, USA; amorris4@wisc.edu

**Keywords:** cystic fibrosis, pharmacists, pharmacy services, medication management, medication use burden

## Abstract

Cystic fibrosis (CF) is one of the most common life-threatening, genetic conditions. People with CF follow complex, time-consuming treatment regimens to manage their chronic condition. Due to the complexity of the disease, multidisciplinary care from CF Foundation (CFF)-accredited centers is recommended for people with CF. These centers include several types of healthcare professionals specializing in CF; however, pharmacists are not required members. The purpose of this study was to identify the outpatient care needs of people living with CF that pharmacists could address to improve their quality of care. Healthcare members from a CFF accredited center and pharmacists were recruited to participate in semi-structured, audio-recorded interviews. Prevalent codes were identified and data analysis was conducted, guided by the systems engineering initiative for patient safety (SEIPS) model. The objective was to understand the medication and pharmacy-related needs of patients with CF and care team perspectives on pharmacists providing support for these patients. From the themes that emerged, pharmacists can provide support for people living with CF (medication burden, medication access, medication education) and the CF care team (drug monitoring and adherence, prior authorizations and insurance coverage, refill history). Pharmacists are well-positioned to address these difficulties to improve quality of care for people living with cystic fibrosis.

## 1. Introduction

Cystic fibrosis (CF) is one of the most prevalent chronic and fatal genetic diseases, affecting approximately 70,000 people worldwide and 30,000 in the United States alone [1,2]. CF is a progressive, multisystem disease that primarily affects the respiratory and digestive systems as well as the pancreas, liver, and reproductive system [1]. CF is an incurable autosomal recessive disorder caused by mutations in the CF transmembrane conductance regulator (CFTR) [3]. The CFTR transports chloride and sodium ions in and out of epithelial cells which controls the movement of water in tissues of the body [4]. Mutations in the CFTR cause thick secretions of mucus to line several organs in the body such as the lungs, pancreas, digestive system, and reproductive system [4]. Consequently, the thick mucus production puts patients with CF at risk of developing bacterial infections in the lungs [1]. Although advances in treatment and knowledge of CF have extended the median predicted survival age to 47.7 years for individuals born in 2016 (compared to age 42.7 years for those born between 2012 and 2016), patients manage complex, time-consuming, and lifelong treatment regimens [1,5].

Medication management with CF is challenging and burdensome. Patients often use eight or more medications daily with lengthy treatments, such as inhaled antibiotics, that can range 1–3 h each day [1]. CF medications include CFTR modulators, mucus thinners, bronchodilators, antibiotics, anti-inflammatories, and pancreatic enzymes [6]. Patients with CF also need to perform airway clearance techniques which may include the use of a vest and nebulizer treatments [6]. Vest treatment is a high-frequency chest wall compression therapy that loosens mucus in the lungs and can be performed in 30-min sessions two to four times per day. Treatment of comorbidities such as depression, anxiety, and diabetes further complicate CF management [4]. Consequently, CF medication adherence can be as low as 50%, particularly among children and adolescents [1,7]. Poor adherence can lead to negative health outcomes such as exacerbations, hospitalizations, and increased healthcare costs [7].

The complexity of managing CF warrants a multidisciplinary healthcare team. The CF Foundation (CFF) recommends that patients visit accredited centers with specialized healthcare professionals at least four times annually [8]. The CFF requires accredited centers to include healthcare team members such as physicians, nurses, respiratory therapists, dietitians, social workers, and program coordinators [9]. Pharmacists are only listed as recommended healthcare team members [9]. However, CF standards of care in countries such as Australia and Britain consider pharmacists to be vital healthcare team members [10,11,12,13]. There has been limited research exploring how pharmacists in outpatient settings can support people living with CF, which is a missed opportunity to improve the care of these patients. There is an urgent need to increase access to pharmacist-provided outpatient care for people with CF to improve their treatment adherence, medication self-management, and overall health-related quality of life. This study aims to describe works system characteristics for the pharmacists’ role in caring for patients with cystic fibrosis.

## 2. Materials and Methods

### 2.1. Theoretical Framework

The systems engineering initiative for patient safety (SEIPS) 2.0 Model was applied to guide our understanding of medication management and interactions between pharmacists and other members of CF healthcare team [14]. The SEIPS model is the most widely used systems engineering framework for patient and healthcare research and embraces three important principles: (1) A holistic systems-based approach, (2) person-centeredness, and (3) design-driven improvement needs to be person-centered to enhance and improve outcomes. The framework proposes the following components of the work system: Person, organization, tools and technology, environment, and tasks. To explore a collaborative process in which patients, pharmacists, and other members of the CF healthcare team can actively engage in medication discussions, it is necessary to understand the activities (or “work”) that each of these individuals carries out with regard to CF medications.

In the SEIPS framework, person(s) are professional or non-professional individuals and characteristics of those individuals, like age and expertise. Tasks are specific actions within larger work processes, with characteristics such as difficulty, complexity, variety, ambiguity, and sequence. Organizations, in the SEIPS framework, are thought of as structures external to a person put in place by people that organize time, space, resources, and activity. Tools and technologies are objects that people use to do work or that assist people in doing work, and is described by usability, accessibility, familiarity, etc. The SEIPS framework separates environment into two factors: The internal environment (the physical environment—lighting, noise, temperature, etc.,) and the external environment, which are economic, ecological, and policy factors outside an organization.

### 2.2. Setting, Sample, and Recruitment

CF healthcare team member participants were recruited from a CFF-accredited center in an urban city hospital in Western Pennsylvania. Pharmacists were purposefully recruited from independent, inpatient, outpatient, and small-chain pharmacies in an urban city in Western Pennsylvania. The research team worked with the center director to identify study participants. CF healthcare member participants declined compensation and pharmacist participants received a $50 incentive. Verbal consent was obtained from study participants. This study was approved by the University of Pittsburgh Institutional Review Board.

### 2.3. Data Collection

We conducted key informant interviews with stakeholders integral to the CF medication management process. The research team developed semi-structured interview guides using open-ended questions to understand the medication and pharmacy-related issues for people living with CF. Two members of the research team assessed the interview guides and provided feedback to ensure content validity. Appendix A contains the guides used to conduct study interviews with pharmacists and other healthcare team members (such as physicians, nurses, dieticians). A research assistant conducted 20-min, in-person, and audio-recorded interviews with study participants from July to September 2016. Participant demographic characteristics such as age, sex, ethnicity, and race were collected. Interviews were conducted until data saturation was achieved. All interviews were professionally transcribed verbatim. To ensure study rigor and trustworthiness, pilot-tested interview guides were used, and reflective journaling and peer debriefing were completed after each interview.

### 2.4. Data Analysis

The research team reviewed the transcripts for accuracy. Interview transcripts were analyzed to develop a list of codes that represented the main conceptual categories within the data. An initial draft of the codebook was developed by members of the research team. The codebook was later simplified and refined based on the results of the first two rounds of coding. Coding was carried out using NVivo10 (QSR International, Melbourne, Australia), a qualitative data analysis software program that enables multi-coder projects. Interviews were coded by at least one of the coders, with the two coders overlapping on 12 of the interviews. Disagreements in coding were adjudicated jointly by the principal investigator and coders. The interview transcripts were coded to identify prevalent themes. Bi-weekly coding meetings were held to review all codes and resolve any discrepancies. Two coders coded the interviews to ensure interrater reliability and had an average Kappa score of 0.60. After the initial coding process, AM conducted deductive content analysis using the categories described in the work system components of the SEIPS 2.0 theoretical framework. Interview transcripts were reviewed using the SEIPS 2.0 Model by searching for key words, such as “pharmacist”, “benefit”, “patient”, “medication”, or “management”. Responses were classified according to the corresponding work system components of the SEIPS 2.0 model. Within those components, similar topics and ideas were aggregated into constructs. OA reviewed the application of the SEIPS 2.0 model to the data to validate the coding. Any discrepancies or differences in opinion were resolved and consensus was researched before final results were obtained.

## 3. Results

A total of 22 participants were interviewed, including 8 pharmacists, 6 pulmonologists, 6 nurses, and 2 dietitians. Of the 22 participants, 12 (55%) were female, 12 (55%) were aged 50 years or older, 21 (95%) were non-Hispanic and White, 7 (32%) had worked 0–10 years, and 7 (32%) had worked 21–30 years (Table 1). The predominant themes that emerged from this study are discussed within each of the work system components of the SEIPS 2.0 model [14]. Additional verbatim quotes from study participants which elaborate on each construct beyond what is provided in the results section is available in Appendix B.

### 3.1. Person

Most members of the CF healthcare team have thorough experience working with people with CF. Pharmacists have variable levels of expertise in CF and in providing services for people with CF. There is also variation in the setting in which these pharmacists had experience providing service to people with CF.

#### Experience and Expertise Caring for People with CF

Pharmacists experience with CF varied: Two pharmacists described providing pharmacy services to only one to two patients with CF, while three pharmacists reported working with patients with CF for many years. As pharmacists’ experience working with CF varied, so did their understanding of the disease. All pharmacists understood healthcare services for patients with CF to be complex, robust, and requiring of multidisciplinary care, no matter the amount of experience they reported.

*“I’ve dealt with [CF] for like 30 years… From the medication standpoint I think I know pretty much a lot of what their needs are, for the medications they need, and also the insurance coverage and everything that I try to do.”—Pharmacist 8*

### 3.2. Tasks

Tasks that participants identified for pharmacists to perform include medication management, medication education, and medication access.

#### 3.2.1. Medication Management Burden

Any tasks that must be done by patients, members of the CF care team, or pharmacists to maintain patient compliance to their individual medication regimen or treatment plan are considered in medication management. All members of the healthcare team and most pharmacists identified a high treatment burden in people with CF, describing medication management as complex, time-demanding, confusing, and difficult for the patients in their care. Nurses and pharmacists described additional medication management burden for the pediatric CF population.

*“I think adherence, treatment burden. I mean, our patients can have like an hour and a half of inhale antibiotics twice to three times a day. Plus, enzymes with each meal and every snack.”—Nurse 1*

*“I think a lot of them struggle because they have so many meds to take. I think sometimes it can be difficult for them to necessarily be motivated to take all their medications.”—Dietician 1*

#### 3.2.2. Medication Education

All healthcare professionals rely on and advocate for verbal education between a patient with CF and each member of the care team. Pharmacists highlight the importance of specialized, one-on-one counseling, but the frequency by which they believe this counseling should occur and the topics that should be covered by pharmacists varied. Physicians verbally educate people with CF, but primarily rely on nurse educators to supplement initial educational conversations. Nurses educate verbally and provide written materials for people with CF, but describe CF education as a team effort, including dieticians and respiratory therapists as educators. Nurse educators do not rely on community pharmacist involvement to educate patients with CF on their medicines. Members of the existing CF healthcare team highlighted additional educational efforts that needed to be in place for transition from child to adult care.

*“great benefit comes from one on one counseling. We will, as pharmacists here, we will actually get to a patient’s home, counsel them on how to actually use their medication.”—Pharmacist 3*

*“we, the [doctors] may introduce the medication to the patient… but then that gets followed up by our nurse educators… I think they sort of try to reinforce our initial educational efforts.”—Physician 3*

Many physicians stated they were aware that patients and their families want to hear information from the doctor, not other members of the healthcare team. Nurses also felt that patients wanted to hear information from the physician.

*“Parents and patients—I think, want to hear it from the doc, but I think giving them a different perspective from somebody else has some real power.”—Physician 2*

There was unanimous agreement amongst the healthcare providers that verbal counseling should be supplemented by additional education materials. There was a split opinion about the effectiveness of this material, as this content was currently offered as written paper pamphlets. Dieticians also employ pre- and post-testing material during verbal counseling.

*“I think that combined with some of the technology and certainly as I said, the handouts, the pamphlets, paperwork, things like that. Something tangible.”—Pharmacist 5*

*“I think most of the pamphlets go in the garbage.”—Nurse 5*

#### 3.2.3. Medication Access and Insurance Challenges

All healthcare professionals discussed the challenges people with CF face regarding insurance. Prior authorizations were described as a major challenge by the healthcare team, and nurses and pharmacists identified this was something in which pharmacist could assist.

*“definitely access. A lot of the times, patients struggle with getting prior authorization or letters of medical necessity, which oftentimes can delay therapy. Or even maybe as simple as their insurance will only cover the medication if it’s coming from a specialty pharmacy”—Pharmacist 1*

*“If we could get more of a pharmacist, we would… I would love for some of the higher-level prior authorizations to be taken over, too, by the pharmacist.”—Nurse 2*

Physicians and nurses also discussed financial burden for people with CF because of the high cost of medications. Pharmacists also identified medication access challenges regarding the need for one patient with CF to rely on multiple pharmacies (including specialty pharmacy) to obtain all their medications.

*“I think they have challenges in terms of expense, they have challenges in terms of getting them through their insurance, and they have challenges just keeping up with the compliance intake.”—Physician 6*

### 3.3. Organization

The organizational impact on pharmacist involvement in the CF care team was highlighted by study participants.

#### 3.3.1. Awareness of Pharmacy Services

Physicians unanimously reported that they rarely interacted with community pharmacies, and many physicians identified that nurses were the primary point of contact with pharmacists. Most nurses reported they communicate with outpatient pharmacies daily via telephone conversations. Dieticians also report working with pharmacists daily, primarily to overcome insurance barriers.

*“I don’t always necessarily think their knowledge of CF is as vast as it could be, I mean I know it’s a very specialized disease. But most of the time, if you provide them with an explanation, they’re willing to work with you… I’d say I deal with them pretty regularly though. Daily.”—Dietician 1*

Existing communication between the CF care team and pharmacists was described as minimal by physicians. The healthcare team members had some ideas by which this communication could be improved, such as including pharmacists on rounds.

*“It isn’t much. I mean, I’m not going to be judgmental and say the communication is poor. I would just say that there tends not to be much.”—Physician 5*

*“I think it’s not even the pharmacists that we need to improve communication with, but the layers that exist before the pharmacist… when you finally get to that pharmacist, they understand, they know what you’re talking about and it’s a little easier.”—Nurse 4*

#### 3.3.2. Benefits and Drawbacks of Pharmacist Involvement

Pharmacists identified several areas in which they could be helpful, including medication education and discharge counseling, relieving other care team members’ workload by handling drug-related issues, and providing private counseling in community pharmacies to improve adherence.

*“we can provide a different aspect of monitoring care that the physicians then don’t need to do. So all the drug level monitoring, some of the compliance monitoring, some of trying to figure out how to make things taste better, or how to fit them down a G-tube, or how to avoid drug interactions by spacing certain meds away from each other.”—Pharmacist 6*

*“I think the most important benefit is—would be number one, education.”—Pharmacist 3*

The healthcare team members identified several ways in which pharmacists could contribute to overall care for patients with CF, including insurance coverage and drug acquisition, refill history, medication safety, and patient education.

*“It would be particularly useful if there were pharmacists always available who could be doing the tracking and the, making sure that the flow of medications continues through the paperwork and other hurdles.”—Physician 6*

*“As the medication regimens are getting more and more complex, we need the expertise of a pharmacist to help do some of those higher prior authorizations that require a little more knowledge and data to defend”—Nurse 3*

Pharmacists anticipated only a few drawbacks of being a member of the healthcare team that cares for people with CF. The primary concern discussed by several pharmacists was care coordination and the increased complexity required to uphold good communication practices between the care team. Pharmacists also mentioned increased costs for the healthcare system, as pharmacists were not included in the hospital’s existing financial model.

*“I think bringing all parties together so that we’re all working cohesively is probably the biggest challenge.”—Pharmacist 4*

*“If you don’t have robust communication with the nursing staff as well as the physician or collaboration between the three of them, then that can create a serious issue…”—Pharmacist 3*

*“So not any drawbacks that I can think of other than, possibly, I guess, increased cost of the health care system. But in the hopes of the program, or the hopes of the CF pharmacist is to reduce costs elsewhere.”—Pharmacist 1*

Members of the CF care team also identified increased financial burden on the hospital to support pharmacists providing care but were primarily concerned with patients feeling overwhelmed by having to visit with another member of the healthcare team. The care team was also concerned that community pharmacists’ knowledge about CF may be limited to properly support people with CF.

*“Our patients meet with four or five team members in every visit, so inserting yet another person that extends their hours long visit, they just might get tired of seeing so many people.”—Physician 6*

*“The barriers will include, education. Not every pharmacist is trained in CF… and so, there’s issues around patient populations, and disease frequency”—Physician 5*

*“Predominantly money… Resources, limited resources! Space and money. Yeah, and time”—Nurse 3*

### 3.4. Tools and Technologies

Participants discussed the potential for a mobile application to be used as a tool to improve medication adherence for people with CF. Pharmacists also identified the use of social media as a tool to communicate with people with CF.

#### Technologies That Influence Adherence and Prescribing

All healthcare professionals perceived benefit to offering a mobile application for use by people with CF, though there were differing opinions about the content and purpose of the app. In general, pharmacists described the app as a tool to support existing medication management techniques. Physicians thought an app could be helpful for self-management or compliance.

*“I always feel that some sort of knowledge center with that has a wealth of videos, a video library that would be able to, number one show people how to use their medication. Number two, go through the clinical aspects of the medication. Side effects, storage, and stability.”—Pharmacist 3*

*“I think it could be very helpful for, perhaps, keeping them on track for when they’re supposed to take them… as a reminder function, I think it could be very valuable.”—Physician 3*

*“We’ve had different nutrition apps and we’ve asked people to go home and look at things and that doesn’t really happen.”—Dietician 1*

There were some concerns to offering a mobile application, including effectiveness of a mobile app as an educational tool, and how the app could be safely offered in clinic, using a communal device.

*“Well, in the clinic setting that’s hard to do on a pad because it has to be sterilized between patients. And so, we think it’s easier to hand out papers.”—Nurse 2*

*“In terms of whether they would take the time to actually learn the details of the medicines through an app, I’m a little skeptical but it might work.”—Physician 6*

Participants were also concerned that a mobile application may not be appropriate for all ages of people with CF. Pharmacists believe medication education is only successful if patients were reached in their environment, providing education through blog posts or social media, or preparing them to explain CF in their own way.

*“I think that is targeted—it would definitely be for adolescents, and not necessarily our older patients.”—Physician 4*

*“I mean if I’m looking at how old the average patient is, you know, we gotta look where they are. So you would use the data to suggest, ‘Hey they’re probably on Facebook’…you scroll and you’re watching and it’s like, everything is in a video. It’s all video digesting content.”—Pharmacist 2*

In terms of other medication adherence tools, there was interest among physicians to implement compliance-tracking devices to install on the patient’s vest.

*“We are now instituting a way of determining whether patients are using their vest. There are some devices that you can plug the vest into, and so you can tell from the electrical current use whether the device has been turned on or not.”—Physician 5*

### 3.5. Environment

Participants solely discussed external environment factors, including collaborative practice agreements for pharmacists and issues with care team access to complete refill histories.

#### 3.5.1. Collaborative Practice Agreements

Pharmacist participants advocated for their CF care team involvement as decision-makers through collaborative practice agreements. Pharmacists had several ideas about what the partnership between pharmacists and other healthcare professionals could look like, and identified many reasons this would be beneficial to improve health care for people with CF.

*“I think that pharmacists have an opportunity to even go further and be—take part in collaborative practice agreements… where pharmacists could change the therapy without having direct physician oversight.”—Pharmacist 1*

*“We would be a physician extender. So the physician is billing for the services that the pharmacist provides… we touch these patients multiple times a month.”—Pharmacist 2*

#### 3.5.2. Refill History Access

All members of the CF care team rely on refill and dispense histories as their primary method to tracking medication adherence, and supplement refill history knowledge by discussing medication adherence with the patient. Physicians also evaluated lung function tests to supplement refill history and improve their understanding of patient compliance.

*“No, not one particular method… you often can tell by their nonverbals, by the look on their face when you ask them a question… and we have patient fill histories that we can look at.”—Nurse 3*

*“I always discuss it with the patient to see what barriers there are between them adhering to the medication and non-adherence… I think it’s very individual.”—Dietician 1*

## 4. Discussion

Despite the benefits that inpatient pharmacists have made in the care of patients with CF, pharmacists are not required members of the healthcare team at CFF-accredited centers [15,16,17]. The perceptions of CF care team participants about where pharmacists could contribute were reported within the components of the SEIPS model (person, tasks, organization, tools and technologies, and environment) [14]. This study identified specific opportunities for pharmacists to assist with medication challenges experienced by people living with CF, including poor medication adherence, medication counseling, and limited medication access because of barriers such as prior authorizations or insurance coverage. Though pharmacists can provide benefit to CF patient care, members of the CF care team may not fully appreciate the value and contributions pharmacists can provide. Establishing a team of pharmacists with advanced CF expertise may improve care coordination with the CF care team and eliminate some of the issues people with CF face regarding medication management and treatment burden.

Medication adherence due to high medication burden is a serious concern for all members of the CF care team. Study findings identified several medication challenges for people with CF, with the CF care team and pharmacists describing treatment management as burdensome, time-consuming, and complex. The typical medication regimen requires multiple medications in different forms (i.e., inhaled, oral, intravenous) to be taken two to three times each day [1,4,6]. Consequently, medication adherence can be difficult, which has been shown in other studies [1,7]. A recent study found that recognizing the importance of CF medications is a predominant barrier to patient adherence to treatment regimens [18]. The pediatric and adolescent population is especially vulnerable. Previous research has shown that adolescents with CF in particular have low adherence rates which can be attributed to forgetfulness, being too busy, feeling that treatments give them less freedom, and believing that skipping treatment is acceptable [19,20]. To monitor adherence, members of the CF care team frequently consult a patient’s medication refill history, a log that community pharmacists are responsible for maintaining. Results show the CF care team is aware of pharmacist involvement in refill history maintenance.

Further complicating medication adherence, medication access is a significant issue experienced by people with CF; study findings show members of the CF care team are burdened by tasks to improve access for patients in their care. Some insurance providers will only cover medications from specialty pharmacies, which do not always stock CF medications. Insurance companies may also only cover specific medications causing patients with CF to not receive the medications they were prescribed. In addition, medication cost was an identified concern. Participants stated that some CF medications from specialty pharmacies cost thousands of dollars and even those covered by insurance have high copays. The high cost of these medications (i.e., Orkambi^®^, Kalydeco^®^) limits access to patients with CF [21]. In handling medication access challenges, study participants explained that issues with prior authorizations were time consuming and caused delays in patients receiving their CF medications. A previous study found that insurance prominently impacts the health of patients with CF, where patients who had Medicaid or public insurance had a higher risk of death while waiting for a lung transplant than those who had private or Medicare insurance [22]. A previous study shows pharmacists are well-positioned to address prior authorization and insurance coverage challenges that burden existing CF care team workload [23]. Study findings show the CF care team identified pharmacist assistance with this task as a perceived benefit to the organization.

The study identified challenges in care coordination and transitions of care for patients with CF. Pharmacist participants revealed that they are often not included by other healthcare members in the care of patients with CF even though they see the patients more frequently. The coordination of care is often difficult due to the many pharmacies that patients with CF use for their medications. CF care team participants reported they do not expect community pharmacists to have the level of expertise required to properly educate people with CF, knowing that many community pharmacists have little contact or opportunity to experience providing care for a patient with CF. This suggests a community pharmacist’s level of expertise and interaction with nurses and dieticians impacts CF care team awareness of pharmacy services they can provide. Consequently, as shown in a previous study, medication reconciliation needs to be prioritized [24]. Pharmacists are easily accessible medication experts that can provide clinical services to people with chronic conditions such as CF more frequently than other healthcare professionals [25,26,27].

Pharmacists are in the community and can provide counseling and education to people living with CF about their medications or address any barriers. Previous research has shown that pharmacists involved in CF care have provided patient education on medications and treatment management, monitored drug-drug interactions, and detected appropriate medication dosing [15]. Other benefits of pharmacists included in CF care in the inpatient setting include improving medication monitoring, communication with the multidisciplinary healthcare team, and efficient use of resources when caring for patients [16,17]. Due to the complexity of CF treatment regimens, participants recommended that it would be beneficial for the pharmacist to create medication schedules for patients with CF to avoid drug interactions. Participants also stated that pharmacists could assess adherence in people with CF by reviewing their medication list and addressing any issues that arise. In addition, participants recommended that pharmacists address insurance issues encountered by patients with CF. Outpatient pharmacists within CFF-accredited centers will be well-positioned to assist patients to navigate insurance issues with prior authorizations, medication access, and cost. Consequently, this would relieve the burden from the healthcare members at the CFF accredited center and patients with CF. Pharmacists can also provide medication education to CF patients. Results from this study suggest that CF patients prefer online learning materials or interactive technology such as tablets or mobile applications. Previous studies have used smartphone applications and telehealth to improve adherence in patients with CF and were shown to be feasible and acceptable [28,29]. Consequently, innovative methods using technology should be implemented to deliver education to patients with CF.

### Limitations

Healthcare members were recruited from one CFF-accredited center, so the results may not generalizable to all regions. Pharmacists who were knowledgeable about CF were also recruited to participate in interviews which creates selection bias and cannot be generalizable to all pharmacists.

## 5. Conclusions

People living with CF experience many medication and pharmacy-related challenges such as high medication burden, medication access, cost, insurance coverage, and care coordination. Although pharmacists are not required members of the healthcare team for CFF accredited centers, this study identified many benefits of having outpatient pharmacists support patients with CF. Pharmacists can help relieve the medication burden for patients with CF by creating schedules that avoid drug interactions. Additionally, pharmacists can assist with assessing drug monitoring and adherence, and assist with insurance issues.

## Figures and Tables

**Table 1:** Participant Demographics (n = 22).

Characteristics	No. (%) ^1^ n = 22
Gender	
Women	12 (55)
Men	10 (45)
Age	
<30 years	3 (14 1)
30–49 years	7 (32 1)
≥50 years	12 (55 1)
Race	
White/Caucasian, Non-Hispanic	21 (95)
White, Hispanic	1 (5)
Role	
Nurse	6 (27 1)
Pulmonologist	6 (27 1)
Registered Dietician	2 (9 1)
Pharmacist	8 (36 1)
Years Worked	
0–10 years	7 (32)
11–20 years	4 (18)
21–30 years	7 (32)
30+ years	4 (18)

^1^ Because of rounding, percentage may not total 100.

## Appendix A

### Appendix A.1. Pharmacist Interview Guide

**A. Introductory Questions about Cystic Fibrosis (CF)**

1. Can you describe what do you know about CF?
2. What do you know about the healthcare services needed for patients with CF?
3. Have you had experiences providing pharmacy services for patients with CF?
  If Yes:
  - Please describe the pharmacy services that were provided.
  - Were there specific medication use challenges that these patients experienced?
  - Were there specific pharmacy-related problems they experienced? (i.e., access to medication, insurance, etc.)
  - Were these able to be addressed?
  If No:
  - Describe the pharmacy-related needs or problems of CF patients. (May probe for issues related to access to medications, insurance issues, etc.)
  - What recommendations do you have for addressing these pharmacy-related issues?
4. What do you think are the benefits or drawbacks of pharmacist involvement in the care of patients with CF?
5. Can you describe how pharmacists could potentially partner with other healthcare professionals to improve care for patients with CF?

**B. Support for People Living with CF**

We would like to know more about how pharmacists can support people living with CF.

1. From your experience, what common difficulties do patients have when taking medications for chronic conditions?
2. Can you describe possible ways pharmacists could assist patients with CF?
  (How do you think pharmacists could be equipped to support patients with CF?)
  - Do you think pharmacists can support people living with CF? If so, how?
    In what ways can community pharmacists uniquely support people with CF?

**C. Preferences for Medication Education**

We would like to know more about how you think people living with CF should be educated about their medications.

1. How do you think a patient using medication for CF should be educated on his/her condition and medication regimen?
  - How often do you think a patient using medication for CF should be educated about taking his/her medications correctly?
2. If you have provided medication education for patients with CF, what types of educational materials have you used?
  If No: What types of education materials have you used with patients with other types of chronic conditions?
3. What types of education materials do you think would be most helpful for providing medication education for patients with CF?
4. Assuming patients want information about their medications, how do you think patients prefer to receive education about their medications?
  Probe for different educational methods (one-on-one counseling with the pharmacist, written materials/pamphlets, interactive technology like on an iPad, videos on the Internet or TV, using an app on a smartphone or tablet)
5. Do you think patients with CF would be interested in using an app, like on a smartphone or tablet, to learn about his/her medicines and self-management? If so, why?
  If Yes:
  - How do you think an app could help patients with CF to learn about their medicines and self-management?
  - What features and content do you think would be beneficial to the app design?
6. Of the methods we discussed, which do you believe is the most effective method for delivering education to patients at a community pharmacy?

**D. Pharmacist Counseling**

We will now ask you about providing medication counseling for people living with CF.

1. How do you feel about talking to patients with CF about their medications?
2. Can you describe potential ways that pharmacists could be better equipped to provide medication counseling for people living with CF?
3. How do the elements of the pharmacy (waiting area, space) facilitate or impede counseling to patients with CF?
  (If not discussed) Please describe any changes you believe would help.
4. Can you describe other ways to engage younger CF patients in medication use discussions?

Do you have anything you’d like to add before we end?

### Appendix A.2. Other Healthcare Team Members Interview Guide

**A. Introductory Questions**

1. Tell me about what you do in your CF care center.
2. Describe the pharmacy-related needs or problems of CF patients. (May probe for issues related to access to medications, insurance issues, etc.)
3. What kinds of challenges do you think that CF patients face regarding the use of CF medications?
4. What types of medications or treatments seem to be especially hard for CF patients to administer or adhere to correctly?
5. What method(s) do you use to assess if patients are adhering to their medication regimens?
6. What recommendations do you have for addressing these medication or pharmacy-related issues?

**B. Medication Education**

We would like to ask you about providing adolescent or young adults (AYA) with CF information about their medicines.

1. Have you provided any form of medication education to patients with CF?
  - If Yes: Please describe what information was provided (i.e., drug information, dosing, side effects, etc.) and how often you provided medication education.
  - What steps do you take to make sure the patient with CF has understood the medication information you provided?
2. How well do you think that patients with CF are educated on their medications?
  - How can their knowledge about medicines be improved?
3. Who do you think should be educating patients with CF on their medications? (Probe for doctor, pharmacist, nurse, etc.)
4. Assuming patients want to learn about their medications, how do you think patients with CF prefer to learn about their medications? (Probe for one-on-one counseling with the pharmacist/doctor/nurse, written materials/pamphlets, interactive technology like on an iPad, videos on the Internet or TV, using an app on a smartphone or tablet)
5. Do you think patients with CF would be interested in using an app, like on a smartphone or tablet, to learn about his/her medicines and self-management? If so, why?
  - If Yes: How do you think an app could help patients with CF to learn about their medicines and self-management?
  - What features and content do you think would be beneficial to the app design?
6. Are there any other ways that you can think of that would be helpful for patients with CF to learn about their medicines?

**C. Experiences with Outpatient, Specialty, and Community Pharmacists**

We would now like to ask you some questions about your experiences with outpatient and community pharmacists.

1. How often and in what practice setting (i.e., in-patient, outpatient, community pharmacies such as Giant Eagle, Rite Aid, CVS, Walgreen’s, etc.) do you interact with pharmacists involved in the care of patients with CF?
2. How would you describe the communication between pharmacists and other CF healthcare team members?
3. Can you describe any possible ways to improve communication between the healthcare team members and pharmacists?
4. How do you think pharmacists can support the other members of the healthcare team providing care for people with CF?
5. What challenges with treatment and medications do you think pharmacists could possibly address for patients with CF?
6. Do you think there are any barriers to pharmacist involvement in the care of patients with CF?
  - If so, what kinds of barriers?
7. Do you think there would be any benefits by implementing outpatient pharmacy services in your CF center?
  - If so, what benefits?
8. Do you think there are there any barriers to implementing outpatient pharmacy services?
  - If so, what kinds of barriers?
9. Do you think community pharmacists can uniquely support patients with CF? (Pharmacists practicing at Giant Eagle, Rite Aid, CVS, Walgreen’s, etc.) If so, how?

Do you have anything you’d like to add before we end?

## Appendix B

**Table A1:** Themes, subthemes, and verbatim quotes from participant interviews.

Work System Components and Themes	Additional Verbatim Quotes
Person(s)	
Experience and Expertise Caring for People with CF	*“CF is an autosomal recessive disorder essentially where, in a nutshell, you have these CFTR mutations where secretions in different mutli-organ systems become thick… oftentimes leading to lung transplant later on down the road. But it also affects the liver, pancreas, and then also male reproductive systems. So it’s multi-organ disease.”—Pharmacist 1* *“I’ve been able to provide probably only to about two patients that we’ve had with CF—well actually, one with CF”—Pharmacist 3* *“I do cover the pulmonology service on the floors. I do all their pharmacokinetics for all their levels, I help them pick the antibiotics, I make sure all their meds are right, I help with their prescriptions, writing them and whatever, if they ever need me to go in and talk to them about something, I certainly would be able to do so”—Pharmacist 6*
**Tasks**	
Medication Management Burden	*“the challenging administration options for the administration options for the medications as most of them are inhaled, oftentimes have to do them 3 times a day—to 2 times a day… so it takes up a large portion of these patients’ time”—Pharmacist 1* *“I think Cayston^®^… but it’s a three time a day medication. So, I think even though it only takes three minutes to nebulize, it’s three times a day. I find that patients don’t do medications three times a day. Tobi tends to take between twenty minutes and a half an hour to nebulize… even though it’s only every other month, they tend to struggle in those months because it’s an added medication to their day. They seem to be able to get Pulmozyme^®^ in, although when they travel it’s hard because it’s a refrigerated medication. And then HyperSal^®^ is twice a day and it makes you cough, so—and it tastes bad, so some of the patients don’t want to use the Hyper-Sal because it makes you cough.”—Nurse 4* *“I think that one of the main difficulties is just remembering to take the medication… a lot of times patients can get busy and forget to take those dosing of the medications. I think sometimes too, particularly with antibiotics, patients start feeling really well and they decide not to take their medications after they start feeling well. And then on the flip side of things, you can also have patients feel not so well, and they think that their medications are contributing to their poor state and decide not to take their medications.”—Pharmacist 1* *“I know that there are medication use challenges that they do experience because they are children. They are not as familiar with how to use a nebulizer. It can be challenging to them.”—Pharmacist 3*
Medication Education	*“We don’t necessarily expect any local pharmacy, brick and mortar I’m referring to, to do patient teaching for their meds. It’s too complex, too specialized.”—Nurse 3* *“I think most people learn best from one on one counseling. If you give someone written materials, it’s more likely than not that they will not be read… we’re very careful about education in our clinic. And we have a dedicated person call a nurse educator… and they tend to meet with the families and go over the finer points about the medications.”—Physician 5* *“For our end, with the kind of quiz that we’re doing, that’s actually geared more towards our transition patients, so patients going from teens to adults. I think it’s really important”—Dietician 1*
Medication Access and Insurance Challenges	*“So, the pharmacy said the medication was denied, then we have to reach out to the insurance company and do a prior auth… if the auth is denied, then we do the appeal… if we can’t get the appeal, then we have to have our physician reach out. We don’t handle the enzyme authorizations or the supplements. We have a dietician that does those.”—Nurse 1* *“The drugs can be astonishingly expensive. Some as much as $306,000 a year for Kalydeco for example.”—Physician 5* *“coordinating all the prescriptions, right? There’s a lot of fragmentation, right? They’re going to maybe two, three, four pharmacies to get their medicine. And just managing that is a burden.”—Pharmacist 2*
**Organization**	
Awareness of Pharmacy-Related Needs	*“They can help with anti-microbial management looking at past cultures and… helping physicians and nurses decide which anti-microbial regiments would be best for the patient to—as to not limit our options for future use based on antimicrobial resistance.”—Pharmacist 1*
Benefits and Drawbacks of Pharmacist Involvement	*“It’s just infuriating to find out that we think we gave a patient an antibiotic, which was prescribed, and find out eight days later that they haven’t gotten it yet because of insurance hoops, prior authorizations… They’re little snags, but they result in devastating consequences. So, those are all things that a pharmacist can help us with.”—Nurse 3* *“more education, maybe more safety for the patients, better outcomes for the patient.”—Dietician 2* *“Sometimes we go in and we review their medications when they first come into clinic and we go over what they’re taking. I think if there was a more in-depth conversation.”—Nurse 1* *“I think that our nutritionist would like him [the pharmacist] to help in… helping with patients who are interested in our herbal remedies. And what are the food and drug interactions.”—Nurse 5*
**Tools and Technologies**	
Technologies that Influence Adherence and Prescribing	*“we used to call all the pharmacists and order them, so we had more interaction than we do now. Now with the electronic medical record and electronic e-prescribing, we have much less interaction. And I find that we probably have, maybe some or more delays to patients getting their meds because of that.”—Nurse 2* *“I think that video presentations with graphics followed by a quiz is probably the best way to do it so that they learn and then have to self-reflect and then spit out what the answers are.”—Physician 1* *“I think—specifically for young people, videos, apps… some sort of content like that, they can watch it at their own time, rewatch to understand a missing point. And then make notes and reach out to us like, ‘Hey I watched this, and these are the questions I have.’”—Pharmacist 4* *“I think that we have a lot online now and we have iPads… that’s what kids really like. They want to see something online”—Nurse 3*
**Environment**	
Collaborative Practice Agreements	*“I think that we can help them to streamline some of their therapies, or make adjustments, additions and subtractions, like discontinuations of things as they progress through.”—Pharmacist 7*
Refill History Access	*“Well a lot of the pharmacies are set up, especially with the expensive drugs, it seems they try to track them more because they have a vested interest to do so. So, we’ll get reminder calls.”—Nurse 2* *“[the pharmacists] call us—I think that’s a generous—you know, for refills, they’ll call us, or fax for refills and I think that helps families. Some of the pharmacists call for medication lists because they are kind of keeping an eye on what the family is getting and sending automatic refills to the family.”—Nurse 4* *“The most effective test we have right now is lung function testing. And lung function testing is kind of the final common pathway for all therapies. To say if your lungs are good then you must be doing the right thing.”—Physician 5*
